# Kalamazoo, Michigan, Dental Society

**Published:** 1863-02

**Authors:** J. A. Watling


KALAMAZOO, MICH., *January* 27, 1863.

The Eighth Annual Meeting of the Michigan Dental Association was held at the Kalamazoo House, in the city of Kalamazoo, on the 27th, at 10 o'clock A. M.

The President and Vice-President being absent, the Association was called to order by Dr. H. Benedict of Detroit. Dr. J. Mansfield was then called to the chair.

A quorum for the transaction of business not being present, the association adjourned till 7 o'clock this P. M.

EVENING SESSION.

Dr. J. Mansfield in the chair, and quorum being present, the association proceeded to the transaction of business. Minutes of last annual meeting read and approved. Drs. Benedict and Cahoon were appointed a committee to examine candidates for membership.

The following named persons made application for membership, viz:

Dr. T. R. Perry, Dr. P. S. Grimes, Dr. B. B. Sudworth, Kalamazoo; Dr. H. H. Jackson, Farmingtan; Dr. J. L. Clapp, Grand Rapids.

All of whom were elected members of this association upon the receipt of the report of the committee on examination recommending their election.

The election of officers for the ensuing year postponed until Wednesday next.

An essay on "Dental Fecs and Etiquette," by Dr. H. Benedict, of Detroit, read and ordered printed in the Dental journals.

Adjourned to Wednesday next at 8½ o'clock A. M.

WEDNESDAY MORNING SESSION.

President Dr. J. A. Robertson in the chair. Minutes of yesterday's proceedings read and approved.

The association then proceeded to the election of officers for the ensuing year as follows, viz:

*President*, Dr. H. Benedict, Detroit; *Vice-President*, Dr. G. W. Stone, Albion; *Secretary*, J. A. Watling, Ypsilanti; *Treasurer*, Dr. J. Mansfield, Niles.

Drs. Field, Metcalf, and Clapp were appointed Executive, Examining and Publishing Committee.

Drs. J. A. Robertson, A. P. Metcalf, C. E. Bartlitt, J. Mansfield, G. L. Field, P. S. Grimes and G. W. Stone were elected delegates to the American Dental Association to be held at Philadelphia on the last Tuesday of July, 1863.

Report of Treasurer for the preceding year received, accepted, and ordered to be placed on file.

Report of the Secretary for the preceding year also received and placed on file.

Secretary's bill for preceding year received and ordered paid.

On motion of Dr. Benedict the following resolution was adopted:

*Resolved*, That the yearly dues be reduced to the sum of one dollar, and that back dues be collected at the rate of one dollar per annum.

That the Secretary be instructed to furnish the Secretary of the American Dental Association with the names of all members of this Association who have paid their dues and are in good standing.

On motion of Dr. Metcalf the following preamble and resolution were adopted:

WHEREAS, The prices of dental materials and the cost of living having advanced, therefore—

*Resolved*, That the dentists of this Association should raise and maintain their prices in proportion to the increased expenses in performing their operations.

Essay by Dr. J. Mansfield on "Treatment of Exposed Nerve," read and ordered published in the Dental journals.

Adjourned to 1¼ o'clock this P. M.

AFTERNOON SESSION.

Dr. H. Benedict in the chair.

Dr. S. D. Palmer elected honorary member of this Association.

The remaining portion of the day was spent in miscellaneous business.

Association adjourned to meet in the city of Jackson on the 3d Monday of January, A. D., 1864, 7 o'clock P. M.

J. A. WATLING, *Secretary*.

